# Effects of Substructure Color, Cement Shade, and Aging on the Color Change of Multilayer Zirconia Laminate Veneer Restorations

**DOI:** 10.3390/jfb17080387

**Published:** 2026-08-05

**Authors:** Ebru Binici Aygün, Bilge Turhan Bal, Seçil Karakoca Nemli, Merve Bankoğlu Güngör

**Affiliations:** Department of Prosthodontics, Faculty of Dentistry, Gazi University, Ankara 06490, Türkiye; bturhan@gazi.edu.tr (B.T.B.); secilkarakoca@gazi.edu.tr (S.K.N.); mervegungor@gazi.edu.tr (M.B.G.)

**Keywords:** multilayer zirconia, laminate veneer, color change

## Abstract

The purpose of the present study was to evaluate the effects of zirconia material type, substructure color, cement color, and aging (before and after aging) on the color change of laminate veneers (LVs) prepared from different multilayer translucent zirconia ceramics. LV preparation was performed on a phantom tooth, and the preparation was digitized to produce resin abutments in two shades (light: A1/B1; medium: A2/A3) to simulate different tooth colors. LVs were designed using dental design software and fabricated from three multilayer zirconia ceramics (multilayered super-high-translucent 5Y-TZP zirconia, multilayered high-translucent 4Y-TZP zirconia, and multilayered ultra-translucent 5Y-TZP zirconia), resulting in 12 experimental groups (*n* = 10). They were then cemented with either clear or white resin cement and subsequently subjected to 10,000 cycles of thermal aging. The color parameters were measured at three time points (before cementation, before aging, and after aging), and the color change values (ΔE_00_) were calculated. The data were statistically analyzed, and the results were compared with the perceptibility threshold (ΔE_00_ = 0.8) and the clinical acceptability threshold (ΔE_00_ = 1.8). Four-way ANOVA revealed an interaction among zirconia material, substructure color, cement shade, and measurement time (*p* = 0.02). Color change values after cementation (ΔE_00_-1) across all groups showed a statistically significant difference between the white and clear cement groups. Before aging, the color change values observed in the 4Y-TZP (DD Cube One ML)-medium abutment–white cement (ΔE_00_-1 = 1 ± 0.2) and 5Y-TZP (Katana UTML)-medium abutment–white cement (ΔE_00_-1 = 1.6 ± 0.7) groups were above the perceptible threshold value but were clinically acceptable (0.8 < ΔE_00_ < 1.8). The color change values observed after aging (ΔE_00_-2) across all experimental groups were clinically unacceptable (ΔE_00_ > 1.8). The color changeof ultra-translucent zirconia LVs was influenced by zirconia material type, cement shade, substructure color, and aging, with the effect of the zirconia material itself being less pronounced. Aging further reduced color stability, resulting in increased ΔE_00_ values and clinically unacceptable color differences.

## 1. Introduction

Laminate veneers (LVs) are conservative esthetic treatments that can be applied with minimal or no tooth preparation [1,2]. They are indicated for anterior teeth with discoloration, diastemas, fractures, wear, malposition, lingual positioning, and dental defects [3]. Recently, feldspathic, leucite, and lithium disilicate ceramics have been extensively employed in restorative applications for LVs [4]. However, the search for alternative restorative materials for LVs continues. Zirconia ceramics, despite their biocompatibility and superior mechanical properties, were previously not preferred for LVs due to their opaque color [1,5]. To overcome the disadvantages of this opaque appearance, highly translucent and shade-gradient zirconia materials have been developed [4,6,7]. Moreover, with the rapid advancement of adhesive techniques, new-generation translucent zirconia ceramics (4Y-TZP and 5Y-TZP) have become a viable option for LVs [2,8]. 4Y-TZP exhibits higher translucency than the older zirconia generation (3Y-TZP) while essentially maintaining its mechanical properties [9,10]. 5Y-TZP provides the highest translucency and superior esthetic performance due to its higher cubic-phase content; however, its mechanical properties are lower than those of 4Y-TZP [10,11]. Furthermore, when these ceramics are fabricated in a shade-gradient structure, they replicate natural tooth structure through graded translucency and color, exhibiting higher translucency in the incisal region and increased chroma and opacity toward the cervical area. The gradient design, achieved by combining different zirconia generations or by adding pigments [1,12,13], supports natural esthetics, with clinical studies reporting high survival rates and low fracture risk for ultra-thin zirconia veneers [4,8,14]. The optical properties of zirconia ceramics can be influenced by several factors, including the type of zirconia, material thickness [15,16,17], type of cement [2], and shade of cement [2,18], underlying tooth color [9,17,19], and aging procedures [20]. Although the high translucency of zirconia LVs provides a natural tooth appearance, their reduced thickness and limited masking ability may pose clinical challenges [15,21]. These materials allow light transmission through the restoration, thereby enabling the color of the underlying substructure or natural tooth structure to influence the final restoration color significantly [9,20,22,23]. Therefore, the shade and type of resin cement used during cementation play a critical role in determining the final color of zirconia restorations [9,24,25]. Additionally, color changes may occur over time due to intraoral conditions. When zirconia ceramics are exposed to the oral environment for prolonged periods, a phase transformation process known as low-temperature degradation (LTD) may occur, initiating at the surface and gradually progressing into the bulk material [26,27]. Contact with water, water vapor, or body fluids can induce a slow and spontaneous tetragonal-to-monoclinic phase transformation in zirconia [28,29]. Because monolithic zirconia lacks a veneering porcelain layer, it is directly exposed to the oral environment, which may further promote LTD [26]. This aging process has been reported to affect the optical properties of zirconia, leading to changes in translucency and color stability over time [30]. Nevertheless, emerging evidence suggests that the clinical relevance of low-temperature degradation (LTD) is limited, particularly for recently developed zirconia materials with improved phase stability. Park [31] reported that LTD treatment did not significantly affect the color, translucency, or hardness of multilayered zirconia. Furthermore, Ban [32] noted that the phase transformation rate of zirconia is highest at 200–300 °C, a temperature range described as “low” only in the context of industrial zirconia testing but far beyond clinically relevant conditions for dental restorations. Accordingly, LTD is currently regarded primarily as a theoretical and materials science concern rather than a major clinical drawback of contemporary dental zirconia. This perspective is also supported by the favorable clinical performance of zirconia restorations. Larsson and Wennerberg [33] reported a cumulative 5-year survival rate of 95.9% for tooth-supported zirconia-based crowns, while long-term clinical results have demonstrated favorable survival outcomes over follow-up periods of approximately 10 years [34]. The existing literature provides limited information regarding the effects of substructure and cement shade, as well as aging-related changes, on the color of multilayer zirconia LVs. Accordingly, this in vitro study aimed to investigate the effects of zirconia material type, substructure color, cement shade, and measurement time points (before and after aging) on color change in different multilayer zirconia LVs. The null hypothesis was that the type of zirconia material, substructure color, cement shade, and aging would not result in statistically significant differences in the color change of multilayer zirconia LVs.

## 2. Materials and Methods

**Figure 1:**
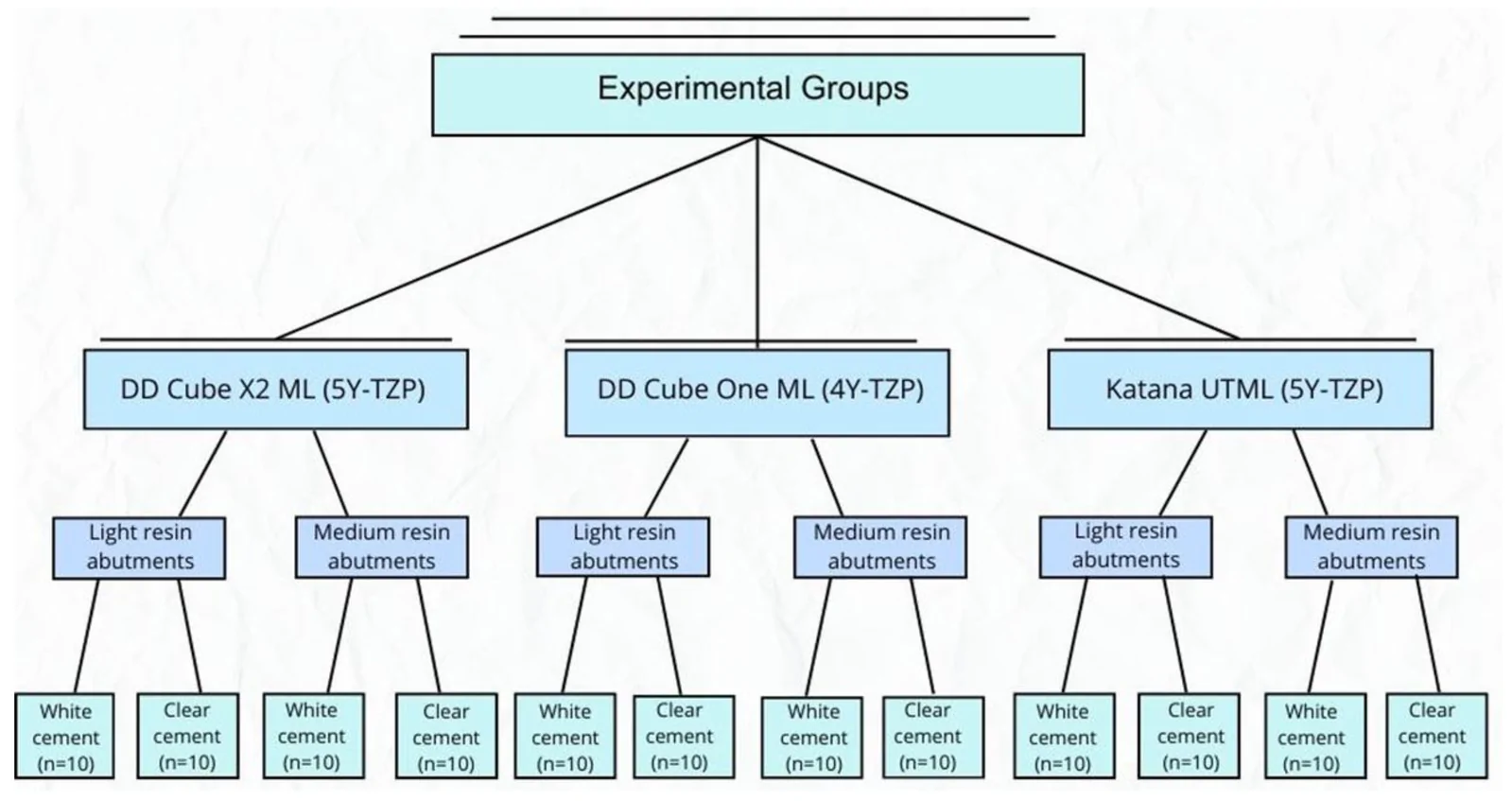
Experimental groups.

This study aimed to investigate the effects of zirconia type, substructure color, cement shade, and measurement times (after cementation and after aging) on the color change in LV restorations fabricated from different multilayer translucent zirconia ceramics. Multilayer super-high-translucent 5Y-TZP zirconia ceramic (Dental Direkt Cube X2 Multilayer, Dental Direkt GmbH, Spenge, Germany), multilayer high-translucent 4Y-TZP zirconia ceramic (Dental Direkt Cube One Multilayer, Dental Direkt GmbH, Spenge, Germany), and multilayer ultra-translucent 5Y-TZP zirconia ceramic (Katana UTML, Kuraray Noritake Dental Inc., Tokyo, Japan) were tested. Additionally, clear and white shades of resin cements, light (A1/B1) and medium (A2/A3) shades of 3D-printed resin abutments, and transparent glaze material were utilized. A total of 12 experimental groups were generated, with 10 specimens (*n* = 10) in each group, as summarized in Figure 1.

**Figure 2:**
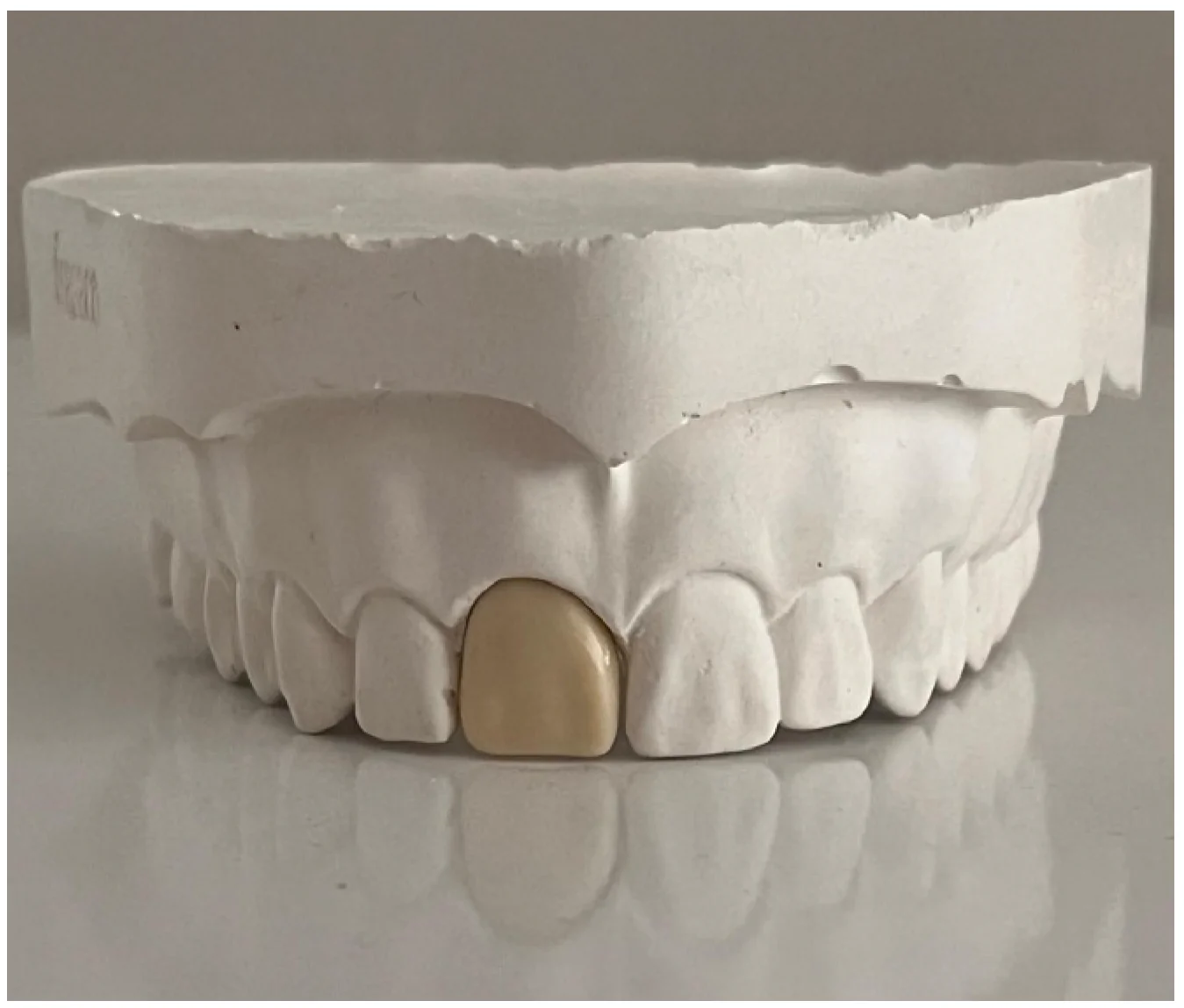
Study model containing an artificial maxillary right central incisor.

The brand names, manufacturers, and lot numbers of all materials used in the study are presented in Table 1. A phantom maxillary right central incisor (Frasaco GmbH, Tettnang, Germany) was isolated and placed into a silicone impression mold (Frasaco GmbH, Tettnang, Germany). Hard dental stone (Zhermack Elite Rock, Zhermack SpA, Badia Polesine, Italy) was poured into the silicone mold to obtain a study cast (Figure 2).

**Figure 3:**
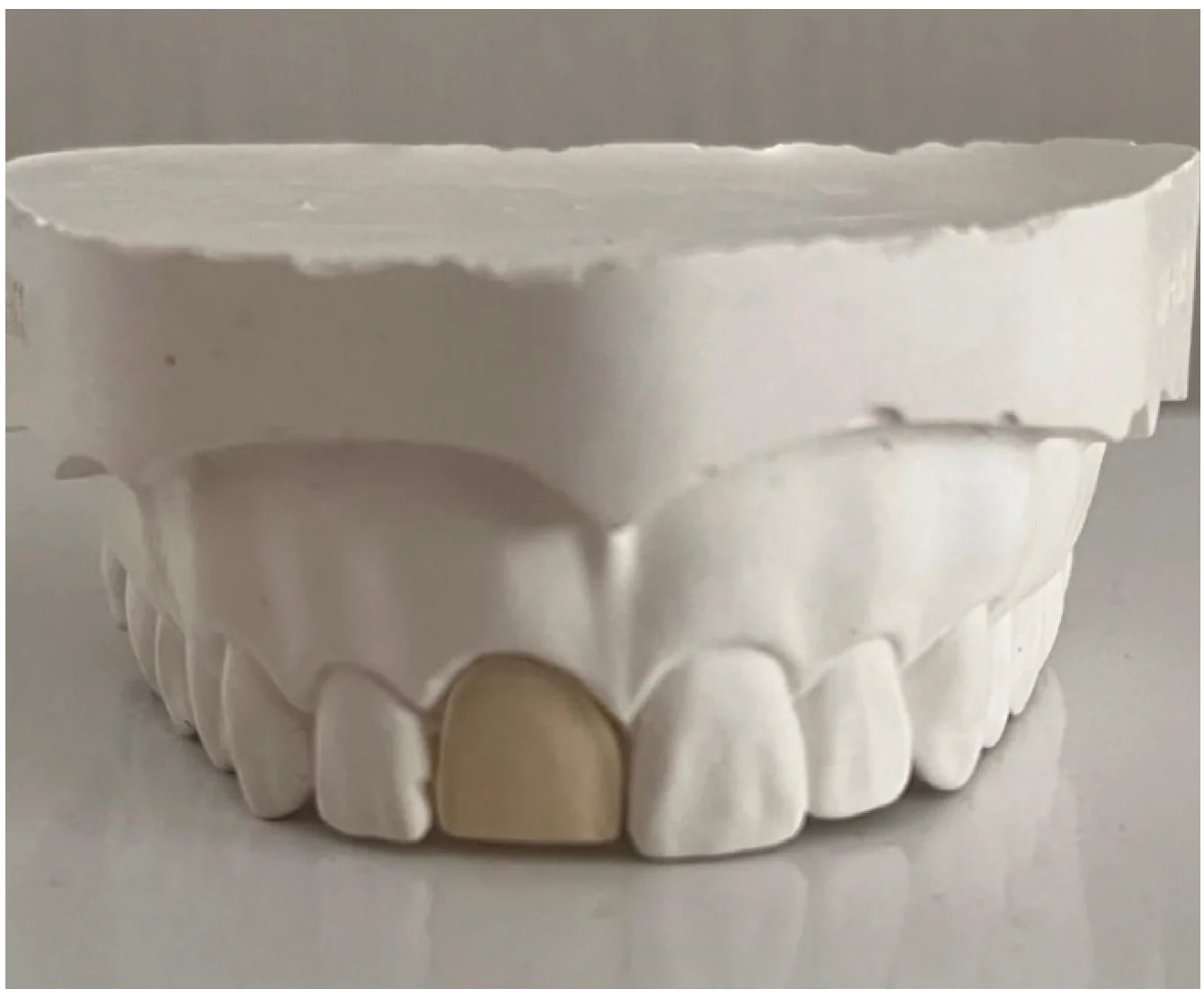
Artificial maxillary right central incisor prepared for laminate veneer restoration.

The LV preparation was performed on the phantom maxillary right central incisor, ensuring a minimum recommended thickness of 0.4 mm and uniform reduction from all surfaces. The marginal finish line was prepared with a 0.4 mm mini chamfer, while the incisal edge configuration was designed as a butt joint (Figure 3).

**Figure 4:**
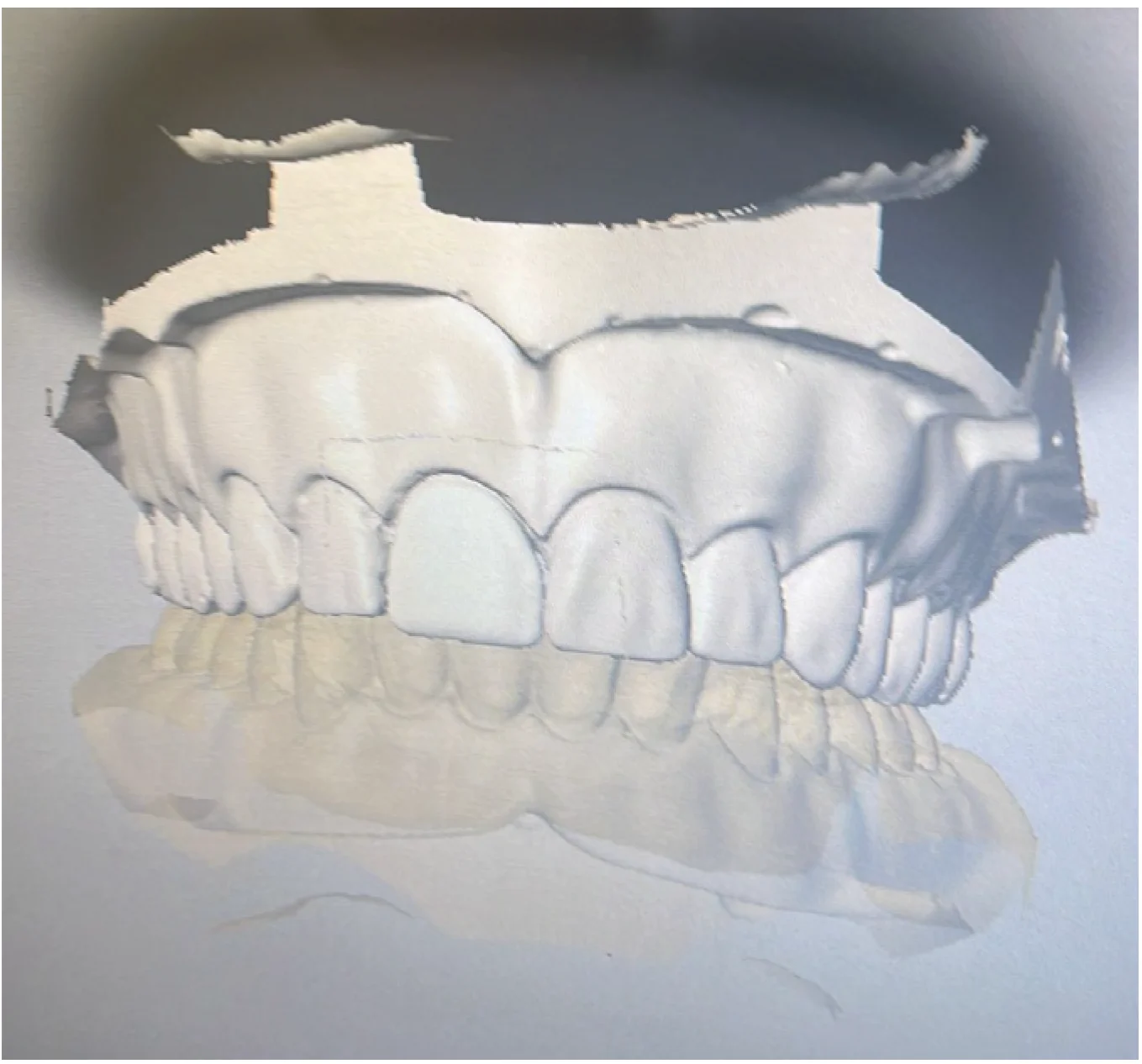
Three-dimensional virtual model and laminate veneer restoration design.

**Figure 5:**
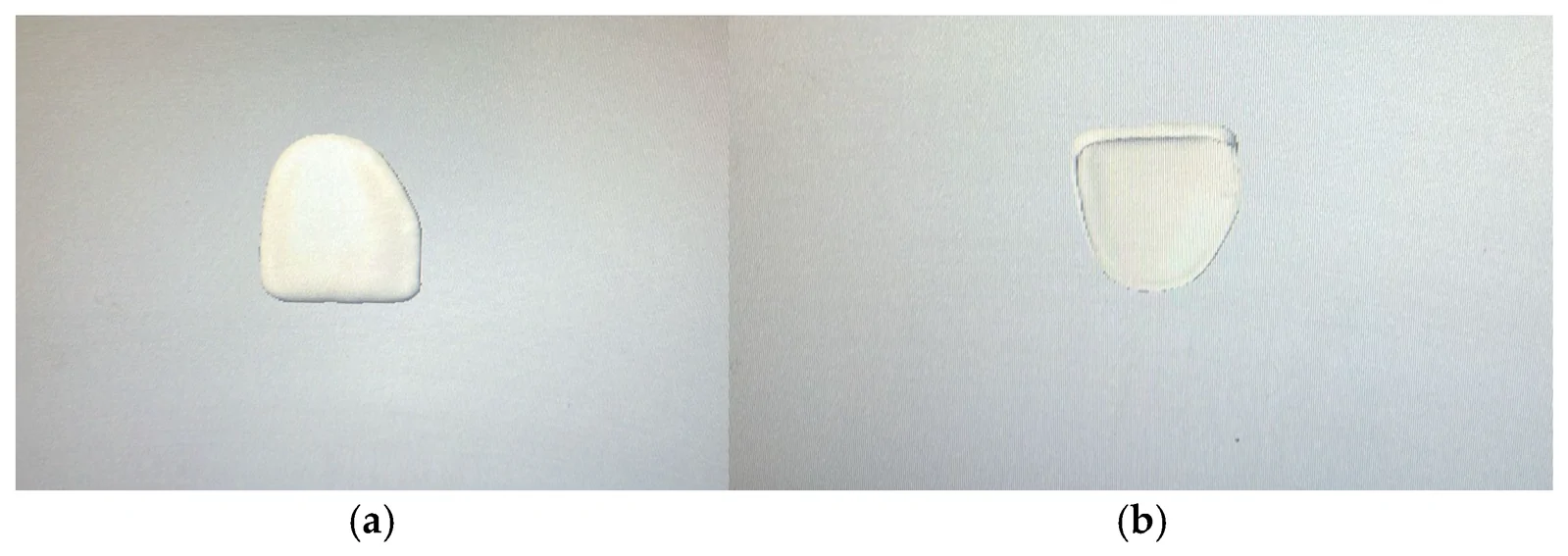
Design of LV restorations: (**a**) Buccal view, (**b**) lingual view.

The study model incorporating the prepared phantom tooth and the opposing cast was mounted on a semi-adjustable articulator (Stratos 200, Ivoclar Vivadent, Schaan, Liechtenstein). The prepared phantom tooth was scanned with the Ceramill Map DRS intraoral scanner (Amann Girbach GmbH, Pforzheim, Germany) to obtain a three-dimensional model. Resin abutments were then fabricated from 3D-printed resin (PowerResins Temp resin, DentaFab, Istanbul, Türkiye) in two colors, Light (A1/B1) and Medium (A2/A3), using a 3D printer (Dentafab Sega 3D printer, DentaFab, Istanbul, Türkiye). After printing, the specimens were sequentially processed utilizing a washing device (DentaWash, DentaFab, Istanbul, Türkiye) and a curing unit (DentaCure, DentaFab, Istanbul, Türkiye) to complete the fabrication of the resin abutments. The fabricated resin abutments were placed into the study model. The models, previously mounted on the articulator, were scanned using an extraoral scanner (Dental Wings 7Series, Dental Wings Inc., Montreal, QC,Canada) to obtain three-dimensional virtual models (Figure 4). The laminate veneer restorations were then designed using dental design software (DWOS, version 9.2, Dental Wings Inc., Montreal, QC, Canada) (Figure 5).

The designed LV restorations were milled from three different zirconia disks in shade A1 using a milling unit (Redon Hybrid, Redon Technology, Istanbul, Türkiye) and sintered according to the manufacturer’s recommended protocol. Following sintering, a glaze procedure was applied to the restoration surfaces. An appropriate amount of transparent glaze powder (Dentsply Ceramco, York, PA, USA) was homogeneously mixed with liquid and applied as a thin layer onto the restoration surfaces. The restorations were then fired in a glaze furnace (Phoenix Quick Cool, Dentsply Ceramco, York, PA, USA). The finished zirconia laminate veneer restorations were seated on the model, and their marginal fit was carefully evaluated. The cementation surfaces of the restorations were cleaned using an ultrasonic cleaner. Subsequently, a ceramic primer was applied to the internal surfaces of the restorations and a tooth primer to the resin abutment surfaces. Resin cement was then applied to the internal surfaces of the restorations, which were carefully seated onto the resin abutments. After 3–5 s of preliminary light polymerization using a VALO Cordless LED light-curing unit (Ultradent, South Jordan, UT, USA), excess cement was removed. Final polymerization was performed for 10 s from each surface using the standard mode (irradiance: 1000 mW/cm^2^; wavelength range: 385–515 nm). Clear and white cement shades were used in accordance with the manufacturer’s recommendations. The cemented specimens were aged in a thermal cycling device (MOD Dental, Esetron Smart Robotechnologies, Ankara, Türkiye). The procedure was performed for 10,000 cycles in water baths ranging from 5 °C to 55 °C, with a 15 s dwell time and a 5 s transfer time.

Before the color measurements, all specimens were cleaned using an ultrasonic cleaner. The color of each specimen was measured at the middle third of the buccal surface using a spectrophotometer (VITA Easyshade Advance 4.0, VITA Zahnfabrik, Bad Sackingen, Germany). All color measurements were performed within a color measurement box under standardized D65 illumination. A white background was used to minimize the potential influence of the surrounding environment on the measurements. The L*, a*, b*, C*, and H* color parameters of each specimen were measured at three steps: before cementation of the restorations, after cementation of the restorations, and after aging. After measuring the color parameters, the color change values (ΔE_00_-1: color change values after cementation (before aging); ΔE_00_-2: color change values after aging) of the specimens were calculated in two stages using the CIEDE2000 formula [35]. The results were compared with the perceptibility threshold (0.8) and the acceptability threshold (1.8) values [36]. The data analysis for the study was performed using IBM SPSS version 23.0, JASP version 0.12, and RStudio, an Integrated Development Environment for R (version 2021.09.0). Descriptive statistics were presented as mean ± standard deviation (mean ± SD). The conformity of the data to a multivariate normal distribution was assessed using the “mvn” package in R. The effects of zirconia material type, substructure color, cement shade, and measurement time (before and after aging) on color change were analyzed using a 4-way factorial ANOVA. The homogeneity of variances was evaluated using the Levene test, and the Tukey HSD test was applied for pairwise comparisons. The level of statistical significance was set at *p* < 0.05.

## 3. Results

The results of the 4-way ANOVA (Table 2) showed a statistically significant interaction (F(2, 216) = 4.31, *p* = 0.02) among the different zirconia materials, substructure colors, cement shades, and measurement times (before and after aging) on color change values.

The mean color change values, standard deviations, and comparative analyses are presented in Table 3. In all zirconia materials, specimens cemented with clear cement exhibited significantly higher ΔE_00_ values than those cemented with white cement, regardless of substructure color and measurement time (*p* < 0.05). Medium resin abutments generally resulted in lower color changes than light resin abutments, particularly when white cement was used.

After aging, several groups showed a significant increase in ΔE_00_ values compared with measurements before aging (after cementation), indicating that aging adversely affected color stability. This effect was more pronounced in specimens luted with clear cement and in ultra-translucent zirconia materials. Among zirconia types, no consistent statistically significant differences were observed when identical substructure color, cement shade, and measurement time were considered (*p* > 0.05), suggesting that optical changes were primarily driven by cement shade, substructure color, and aging rather than zirconia type alone. White cement and lighter substructure colors provided more stable color outcomes, while aging increased color differences.

**Figure 6:**
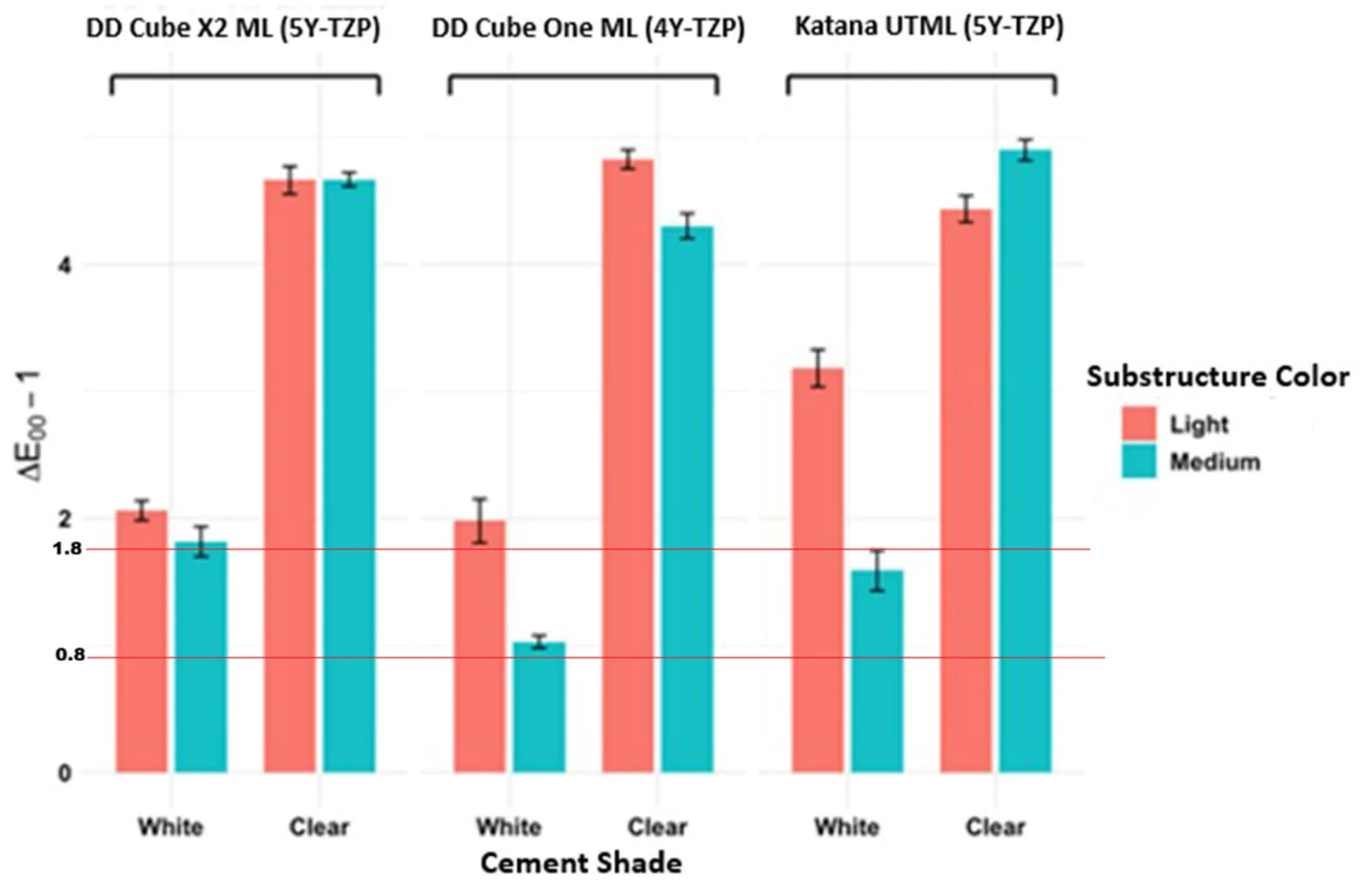
Effect of material type, cement, and substructure color on mean color change before aging (after cementation) (ΔE_00_-1).

**Figure 7:**
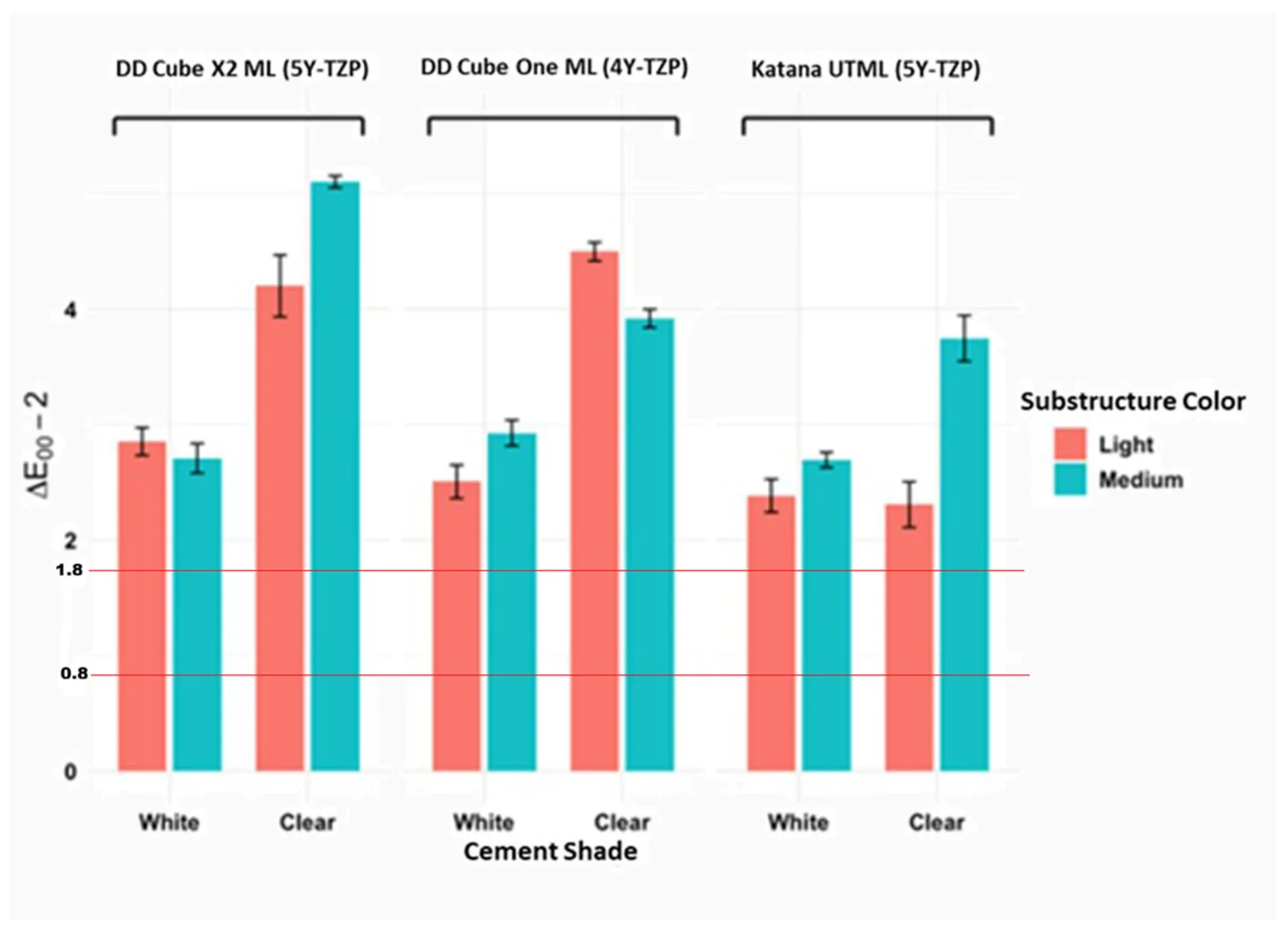
Effect of material type, cement, and substructure color on mean color change after aging (ΔE_00_-2).

Before aging (after cementation), only the DD Cube One ML–medium abutment–white cement and Katana UTML–medium abutment–white cement groups demonstrated perceptible color differences that remained within clinically acceptable limits. In contrast, the DD Cube X2 ML–medium abutment–white cement group was at the limit of clinical acceptability, suggesting a higher risk of visible color mismatch. All other groups exceeded the acceptability threshold (1.8), indicating clinically unacceptable color differences immediately after cementation (Figure 6). Following aging, color differences increased, and all groups exceeded the clinical acceptability threshold (Figure 7).

## 4. Discussion

The effects of zirconia material type, substructure color, cement shade, and measurement time (before and after aging) on the color change values of LVs fabricated from different multilayer translucent zirconia ceramics were investigated in the present in vitro study. According to the 4-way ANOVA results, a statistically significant interaction (F(2, 216) = 4.31, *p* = 0.02) was found among the different zirconia materials, substructure colors, cement shades, and measurement times (both before and after aging) on color change values, and all factors affected the color change values. Thus, the null hypothesis of the study was rejected.

The final color of zirconia LVs is influenced by multiple factors, including the resin cement shade, the substructure color, and the restoration thickness. Previous studies have demonstrated that these variables can significantly affect the color outcomes of high-translucent zirconia materials [1,6,16,22,25]. However, many investigations were performed using nonanatomical specimens fabricated from monochromatic zirconia, flat, uniformly colored backgrounds, or without the application of resin cement between the zirconia and the substructure [1,6,23,25]. Although some studies have evaluated zirconia restorations with anatomical contours [2,7,9], the literature remains limited regarding the combined effects of different substructure colors and resin cement shades. In particular, studies investigating the influence of cement shade on the final color of ultra-translucent zirconia laminate veneers fabricated at minimal thickness are rare. Moreover, although the masking ability of ceramic materials from different manufacturers has been assessed across various thicknesses and substructure types, definitive and consistent conclusions have not yet been established [2,5,17,22]. According to studies and manufacturer recommendations, 0.4 mm thick laminate veneers can be fabricated from ultra-translucent multilayer zirconia [1,8]. In this study, 0.4 mm thick laminate veneers were produced using three zirconia materials: DD Cube X2 ML (5Y-TZP, super-high-translucent multilayer zirconia), DD Cube One ML (4Y-TZP, high-translucent multilayer zirconia), and Katana UTML (5Y-TZP, ultra-translucent multilayer zirconia). These materials were selected to compare the optical behavior of commercially available multilayered zirconia ceramics with different yttria contents and translucency levels under standardized experimental conditions. The inclusion of two different 5Y-TZP materials also allowed the possible influence of manufacturer-specific composition, crystalline structure, and processing characteristics on color behavior to be evaluated. To evaluate their effects on the final color of the restorations, two resin cement shades (clear and white) were selected. In addition, to investigate the influence of substructure color and to simulate natural tooth structures, resin abutments were produced by 3D printing using provisional resin materials in two shades: light (A1/B1) and medium (A2/A3). The results were compared with the perceptibility threshold (ΔE_00_ = 0.8) and the clinical acceptability threshold (ΔE_00_ = 1.8) established for dental ceramics according to the CIEDE 2000 system [36]. In previous studies, it was reported that the shade of resin cement and material thickness influence the final color of translucent ceramic materials and that color difference values increased as material thickness decreased [1,6,15,21,25]. Kang et al. [6] investigated the effect of material thickness on the color of highly translucent monolithic multilayer zirconia materials and concluded that a thickness of 1.0 mm is optimal for achieving optimal color accuracy. Similarly, Tabatabaian et al. [21] reported that the thickness of monolithic zirconia ceramics significantly affected the final color of restorations, stating that a minimum thickness of 0.9 mm is required to achieve an acceptable final color. In the present study, the material thickness was kept constant at 0.4 mm, and the effect of thickness on the final color was not evaluated. However, the mean color change values observed in most experimental groups exceeding the acceptable threshold (ΔE_00_ > 1.8) are likely due to the use of a 0.4 mm material thickness.

In this study, two different shades of a 3D-printed resin material, light (A1/B1) and medium (A2/A3), were used as substructures. The results showed that substructure color alone did not demonstrate a statistically significant influence on the final color; however, when combined with different resin cement shades and highly translucent zirconia materials, substructure color significantly influenced the final restoration color. This finding highlighted the need to consider both substructure color and cement shade, particularly when using highly translucent ceramic materials. Previous studies [1,9,22] consistently demonstrated that substructure color and resin cement shade had a clinically significant effect on the final color of highly translucent ceramic restorations, particularly in thin restorations. Several authors have reported that opaque resin cements improved color outcomes when restorations were placed on dark substructures [18,19]. Dede et al. [18] reported that noticeable masking of the final color was achieved only with an opaque C2 shade, whereas other cement shades did not produce comparable masking. Barath et al. [19] further emphasized that dark substructures could be effectively masked only by combining darker ceramic materials with opaque cements, indicating that neither the ceramic nor the cement alone is sufficient to overcome the optical influence of a dark background. Consistent with these findings, the present study showed that lower mean color change values were observed when a medium-colored substructure (A2/A3) was combined with white cement, regardless of the zirconia material used. Although the ΔE_00_ values varied among zirconia groups, the medium-colored substructure and white cement combination consistently yielded lower color changes than light substructure or translucent cement combinations, supporting the masking effect of lighter, more opaque cements. Similarly, Comba et al. [22] reported that both substructure color and cement shade significantly affected translucency and final color and that milky-bright cement produced lower ΔE_00_ values than translucent cement. In their study, color differences in zirconia remained clinically acceptable (ΔE_00_ < 1.8), whereas lithium disilicate restorations frequently exceeded this threshold. In contrast, the present study found that most zirconia groups exhibited ΔE_00_ values above 1.8, likely related to the higher yttria content and increased translucency of the zirconia materials used, which made them more susceptible to substructure and cement color effects. Comparable results were also reported by Miura et al. [9], who investigated 0.8 mm thick high-translucency zirconia crowns and reported ΔE_ab_ values ranging from 1.39 to 4.27, concluding that both substructure and cement color significantly influenced the final color. Similarly, Plengsombut and Palanuwech [7] reported that the abutment material and cement shade had a statistically significant effect on color difference, with opaque cement producing lower ΔEab values on metal abutments than clear or universal cements.

It was reported that monolithic zirconia presented a higher susceptibility to LTD than zirconia core materials [26]. This degradation process adversely affected the esthetic properties of monolithic zirconia restorations over time. In the present study, the mean color changes exceeding the clinically acceptable threshold after aging may be associated with LTD-related surface alterations. Khomprang et al. [12] evaluated rectangular specimens made of IPS e.max CAD and IPS e.max ZirCAD Prime after 30,000 coffee thermal cycles and reported mean color changes (ΔE_ab_) of 4.7 and 4.6, respectively. With perceptibility (PT = 1.2) and acceptability (AT = 2.7) thresholds, both materials exceeded the acceptable limit, and no significant difference was observed between them (*p* = 0.202), indicating that coffee thermal cycling negatively impacted color stability. Despite differences in the materials evaluated and the aging protocols used, these findings are consistent with the results of the present study. Conversely, Ashy et al. [30] investigated high-translucency lithium disilicate and zirconia specimens cemented with clear light-cure or dual-cure resin cements and subjected to 3000 thermal cycles (5–50 °C). After aging, color changes were perceptible in all groups (PT = 1.0), but only the light-cured lithium disilicate group exceeded the acceptability threshold (ΔE_ab_ = 4 ± 1.8; AT = 3.7). No significant difference was observed between ceramic types. These results differ from the present findings, as color changes across all groups exceeded the clinically acceptable threshold (ΔE_00_ > 1.8). This discrepancy may be attributed to fewer thermal cycles, greater ceramic thickness, and the use of clear cements in their study. Mezied [29] compared the color stability of three cubic zirconia materials, Katana UTML, Cercon XT, and Ceramill Zolid FX UT, and lithium disilicate (IPS e.max CAD HT) after 10,000 thermal cycles (5–55 °C). The highest color change was observed in lithium disilicate (ΔE_ab_ = 2.2 ± 0.2), followed by Cercon XT (1.7 ± 0.2), Ceramill Zolid FX UT (1.4 ± 0.3), and Katana UTML (1.4 ± 0.4). Although all color changes were perceptible, they remained within clinically acceptable limits, with Katana UTML showing the highest color stability. Similarly, in the present study, Katana UTML showed lower color changes than the other zirconia materials after aging. However, the color changes in the present study were clinically unacceptable (ΔE_00_ > 1.8), which may be explained by the additional influence of substructure and resin cement materials, both of which were also affected by aging and contributed to the final color alteration.

This study has several limitations. First, the multilayer zirconia laminate veneer restorations were fabricated only at a thickness of 0.4 mm and in the A1 shade. Evaluating different material thicknesses and shades could yield more comprehensive, comparable results. Second, to avoid variations in ceramic thickness caused by the anatomical crown contour, color change assessment was performed only on the labial surface. Third, the absence of sandblasting represents a limitation of this study. Although sandblasting was omitted because of its potential influence on the optical properties of zirconia, its frequent clinical use to enhance bond strength may limit the direct clinical applicability of the present findings. Fourth, the 10,000-cycle thermocycling protocol used in this study represents a relatively limited period of clinical service and may not fully reflect the long-term color behavior of zirconia laminate veneers. Therefore, future studies using extended aging protocols involving 50,000–100,000 cycles are recommended. Also, the aging protocol was limited to thermocycling, and chewing simulation was not performed. Therefore, future studies should evaluate both the optical and mechanical properties of zirconia materials using aging protocols that combine thermocycling with mechanical loading. Future studies are recommended to evaluate color changes not only in the mid-labial region but also in the cervical and incisal areas. Another limitation is that the provisional resin used as the substructure may have aged, potentially influencing the mean color change values. Therefore, further studies on natural teeth are needed.

## 5. Conclusions

1. Zirconia type, cement shade, substructure color, and aging all influenced the color stability of ultra-translucent zirconia LVs; however, when the other variables were standardized, the effect of zirconia type was less pronounced than that of cement shade, substructure color, and aging.
2. The groups cemented with clear shade cement demonstrated greater color changes than those cemented with white shade cement.
3. Aging had a pronounced negative effect on color stability, resulting in increased ΔE_00_ values and clinically unacceptable color differences across all groups.

## Figures and Tables

**Table 1:** Materials used in the study.

Materials	Brand Name	Manufacturer	Lot Number
3D printing resin (Light, Medium)	PowerResins Temp	3BFAB Technology, Istanbul, Türkiye	MD1120240305011
Multilayer super-high-translucent 5Y- TZP zirconia disk (A1)	DD Cube X2 ML	Dental Direkt GmbH, Spenge, Germany	1162004005
Multilayer high-translucent 4YTZP zirconia disk (A1)	DD Cube One ML	Dental Direkt GmbH, Spenge, Germany	7162310016
Multilayer ultra-translucent 5Y- TZP zirconia disk (A1)	Katana UTML	Kuraray Noritake Dental Inc., Tokyo, Japan	ELYXS
Resin cement (clear, white)	Panavia V5	Kuraray Noritake Dental Inc., Tokyo, Japan	000157 (clear) A50018 (white)
Ceramic primer	Panavia V5 Clearfil Ceramic Primer Plus	Kuraray Noritake Dental Inc., Tokyo, Japan	1W0091
Tooth primer	Panavia V5 tooth primer	Kuraray Noritake Dental Inc., Tokyo, Japan	290126
Glaze powder	Dentsply Ceramco	Dentsply Sirona, York, PA, USA	17002651

**Table 2:** Four-way ANOVA results of the effects of different zirconia materials, substructure colors, cement shades, and measurement time (before and after aging) on color change values.

Source	Sum of Squares (SS)	Degrees of Freedom (df)	Mean Squares	*F*	*p*-Value
Zirconia Material	6.47	2	3.24	10.54	<0.001
Substructure Color	0.23	1	0.23	0.75	0.39
Cement Shade	241.68	1	241.68	787.15	<0.001
Measurement time	0.17	1	0.17	0.56	0.46
Zirconia Material x Substructure Color	3.68	2	1.84	6.00	0.00
Zirconia Material x Cement Shade	11.66	2	5.83	18.99	<0.001
Zirconia Material x Measurement time	19.06	2	9.53	31.04	<0.001
Substructure Color x Cement Shade	5.59	1	5.59	18.21	<0.001
Substructure Color x Measurement time	10.19	1	10.19	33.20	<0.001
Cement Shade x Measurement time	27.94	1	27.94	91.01	<0.001
Zirconia Material x Substructure Color x Cement Shade	8.87	2	4.44	14.45	<0.001
Zirconia Material x Substructure Color x Measurement time	3.05	2	1.52	4.96	0.01
Zirconia Material x Cement Shade x Measurement time	3.14	2	1.57	5.11	0.01
Substructure Color x Cement Shade x Measurement time	1.36	1	1.36	4.42	0.04
Zirconia Material x Substructure Color x Cement Shade x Measurement time	2.65	2	1.32	4.31	0.02
Error	66.32	216	0.031	-	-
**Total**	412.06	239	-	-	-
S = 0.1752, R^2^ = %83.9, Adj-R^2^ = %82.2					

**Table 3:** Tukey multiple comparison test results for color change values according to zirconia materials, substructure colors, cement shades, and measurement times (before and after aging).

Zirconia Material Type	Substructure Color	Cement Shade	Measurement Times	
			Color Change Before Aging (Before and After Cementation) (ΔE_00_-1) Mean ± SD	Color Change After Cementation and After Aging (ΔE_00_-2) Mean ± SD
**DD Cube X2 ML** **(5Y- TZP)**	**Light resin abutments**	**White**	2.06 ± 0.34 ^a,A,1,(A)^	2.85 ± 0.54 ^a,A,1,(A)^
		**Clear**	4.66 ± 0.49 ^a,B,1,(A)^	4.45 ± 0.91 ^a,B,1,(A)^
	**Medium resin abutments**	**White**	1.82 ± 0.53 ^a,A,1,(A)^	2.71 ± 0.57 ^a,A,1,(A)^
		**Clear**	4.67 ± 0.25 ^a,B,1,(A)^	5.10 ± 0.23 ^a,B,1,(A)^
**DD Cube One ML (4Y- TZP)**	**Light resin abutments**	**White**	1.98 ± 0.77 ^a,A,1,(A)^	2.51 ± 0.65 ^a,A,1,(A)^
		**Clear**	4.83 ± 0.33 ^a,B,1,(A)^	4.50 ± 0.36 ^a,B,1,(A)^
	**Medium resin abutments**	**White**	1.03 ± 0.22 ^a,A,2,(A)^	2.93 ± 0.50 ^b,A,1,(A)^
		**Clear**	4.30 ± 0.44 ^a,B,1,(A)^	3.92 ± 0.35 ^a,B,1,(B)^
**Katana UTML** **(5Y- TZP)**	**Light resin abutments**	**White**	3.18 ± 0.65 ^a,A,1,(B)^	2.38 ± 0.64 ^a,A,1,(A)^
		**Clear**	4.44 ± 0.47 ^a,B,1,(A)^	2.31 ± 0.88 ^b,A,1,(B)^
	**Medium resin abutments**	**White**	1.59 ± 0.71 ^a,A,2,(A)^	2.70 ± 0.29 ^b,A,1,(A)^
		**Clear**	4.90 ± 0.37 ^a,B,1,(A)^	3.75 ± 0.88 ^b,B,2,(B)^

SD: Standard Deviation. * Within the same zirconia materials, substructure colors, and cement shades, the measurement times (after cementation and after aging) sharing the same lowercase letter do not differ significantly in terms of mean color change values (*p* > 0.05). ** Within the same zirconia materials, substructure colors, and measurement times (after cementation and after aging), the cement shades sharing the same uppercase letter do not differ significantly in terms of mean color change values (*p* > 0.05). *** Within the same zirconia materials, cement shades, and measurement times (after cementation and after aging), the substructure colors sharing the same number do not differ significantly in terms of mean color change values (*p* > 0.05). **** Within the same substructure colors, cement shades, and measurement times (after cementation and after aging), no statistically significant difference was observed in mean color change values among the zirconia materials that share the same capital letter in parentheses (*p* > 0.05).

## Data Availability

The datasets used and analyzed during the present study are available from the corresponding author upon reasonable request.

## Abbreviations

The following abbreviations are used in this manuscript:

3D	Three-dimensional
4Y-TZP	4 mol% yttria-stabilized tetragonal zirconia polycrystal
5Y-TZP	5 mol% yttria-stabilized tetragonal zirconia polycrystal
AT	Acceptability threshold
LV	Laminate Veneer
PT	Perceptibility threshold
UTML	Ultra-Translucent Multilayered
